# Isolation and Transfection of Protoplasts From Maize Mesophyll Cells

**DOI:** 10.21769/BioProtoc.5596

**Published:** 2026-02-05

**Authors:** Lauren A. Higa, Taren Bouwman, Zhi-Yan Du

**Affiliations:** Department of Molecular Biosciences & Bioengineering, University of Hawaii at Mānoa, Honolulu, HI, USA

**Keywords:** Protoplast, CRISPR, Transformation, Maize, Mesophyll

## Abstract

Protoplast systems are widely used in plant research as versatile platforms for studying cellular processes and validating gene editing tools. In maize, they are particularly valuable because stable transformation in immature embryos is slow and labor-intensive, often requiring months to regenerate plants. However, existing protocols often yield inconsistent results in protoplast recovery, transfection efficiency, and viability. We present an optimized protocol for maize mesophyll protoplast isolation and PEG-mediated transfection. Two-week-old etiolated seedlings are processed using vertical cutting, improving the yield and viability of protoplasts. Protoplasts are then immediately transformed with a CRISPR/Cas9 construct after isolation, via PEG4000 with only 10 μg of plasmid DNA, reducing the resource demands of standard methods. Modified washing and storage conditions extend transformed protoplast viability to seven days, enabling longer-term monitoring and expanded downstream analyses. Editing outcomes are quantified by sequencing target sites and calculating efficiency with Cas-Analyzer. This protocol provides a rapid, efficient, and reproducible method for the rapid evaluation of gene editing in maize. This protocol offers a methodology to accelerate agricultural crop studies and broader plant molecular biology.

Key features

• Optimized maize mesophyll protoplast isolation using vertical cutting to improve yield, consistency, and viability.

• Efficient PEG4000-mediated transformation requiring only 10 μg of plasmid DNA for CRISPR delivery.

• Extended protoplast viability up to seven days through modified washing and storage conditions, enabling longer monitoring and analysis.

• Rapid and reproducible gene-editing evaluation via targeted sequencing and Cas-Analyzer quantification.

## Graphical overview


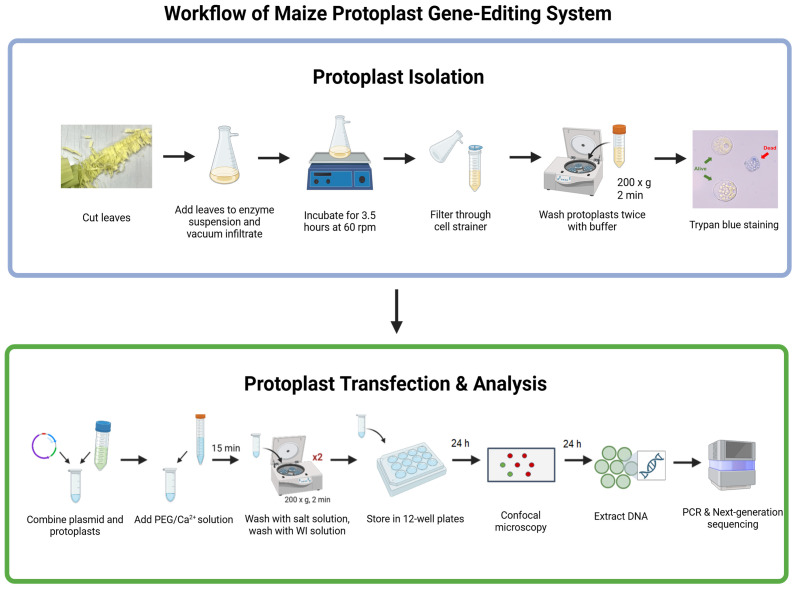


## Background

Genetic engineering of crops is an important strategy for improving yield, nutritional quality, and resilience to environmental stress. CRISPR/Cas9 has become a central tool in this work, yet the efficiency of guide RNAs (gRNAs) can be strongly influenced by chromatin accessibility, GC content, and local nucleotide context [1–4]. Stable transformation in maize remains a slow and resource-intensive process, often taking eight to nine months to regenerate plants and evaluate gene editing events [5]. As such, it is advantageous to evaluate gRNA performance before committing to stable transformation in immature embryos. Protoplast systems provide a practical solution, allowing gRNA activity to be tested in vivo in less than 1 week. However, existing protocols for maize protoplast isolation and transfection often produce variable results in yield, efficiency, and cell viability. For example, seedling growth conditions remain a point of debate, as many studies suggest that etiolated seedlings yield more viable protoplasts [6]. Additionally, cutting methods also influence recovery. While most protocols use horizontal leaf sections, vertical cutting has been shown to release more protoplasts per gram of fresh weight. This improved yield is especially valuable when plant material is limited. Transfection parameters often require high amounts of plasmid, with commonly used protocols requiring 20 μg of plasmid DNA, adding to the burden of plasmid preparation. Many established methods were designed for short-term assays such as luminescence or protein localization, where protoplast viability beyond 6–18 h is not necessary [7–9]. In contrast, gene editing requires cells to remain viable for at least 48 h to capture CRISPR-induced edits. The following protocol incorporates modifications to the methodologies presented in Yoo et al. (2007) and Gomez-Cano et al. (2019) [14,18] to improve yield, reduce DNA input, and extend protoplast viability. Although this protocol is described in the context of gene editing, these improvements can be applied to other monocot systems and adapted for various downstream applications. Additionally, extending protoplast viability allows for longer-term studies of cellular processes, such as protein localization [8,10], protein interactions [7,11], cell signaling [12,13], gene regulation [14], metabolomics [9], and transcriptomics [15]. Some considerations for the utilization of this protocol are that protoplasts cannot currently be regenerated into whole plants, and that edits introduced through non-homologous end joining (NHEJ) are random, so that sequence variants observed in protoplasts may not precisely predict edits recovered after stable transformation [16]. Despite this, characterizing gRNA efficiency in protoplasts is a critical evaluation tool, as high editing efficiency significantly increases the likelihood of generating desirable mutations in subsequent transformations.

## Materials and reagents

**Biological materials**

1. TZI8 maize seeds (Maize Genetics Corporation Stock Center, TZI8)

**Reagents**

1. Bovine serum albumin (BSA) (Millipore Sigma, CAS: 9048-46-8)

2. Calcium chloride (CaCl_2_) (Fisher Scientific, CAS: 10043-52-4)

3. Cellulase (Millipore Sigma, CAS: 9012-54-8)

4. Cetyltrimethylammonium bromide (CTAB) (Fisher Scientific, CAS: 57-09-0)

5. Ethylenediaminetetraacetic acid, disodium salt dihydrate (EDTA) (Fisher Scientific, catalog number: S311-100)

6. Isopropanol, 99.5% (Fisher Scientific, CAS: 67-63-0)

7. Potassium chloride (KCl) (Fisher Scientific, CAS: 7447-40-7)

8. D-Mannitol (Fisher Scientific, CAS: 69-65-8)

9. Macerozyme (Research Products International, CAS: 9032-75-1)

10. 2-morpholin-4-ylethanesulfonic acid (MES) (Fisher Scientific, CAS: 145224-94-8)

11. Magnesium chloride (MgCl_2_) (Fisher Scientific, CAS: 7786-30-3)

12. Polyethylene glycol 4,000 (PEG 4000) (Fisher Scientific, catalog number: AAA1615130)

13. RNase A solution (Fisher Scientific, catalog number: PR-A7973)

14. Sodium chloride (NaCl) (Fisher Scientific, CAS: 7647-14-5)

15. 2-Amino-2-(hydroxymethyl) propane-1,3-diol (Tris-HCl) (Fisher Scientific, CAS: 77-86-1)

16. Trypan Blue solution, 0.4% (Fisher Scientific, catalog number: 15250061)

17. UltraPure^TM^ phenol:chloroform:isoamyl alcohol (25:24:1, v/v) (Fisher Scientific, catalog number: 15593031)

**Solutions**

1. Protoplast buffer (see Recipes)

2. MMG buffer (see Recipes)

3. W5 buffer (see Recipes)

4. WI buffer (see Recipes)

5. PEG solution (see Recipes)

6. CTAB buffer (see Recipes)

7. Enzyme suspension (see Recipes)

**Recipes**

**1. Protoplast buffer**

**Table:** 

Reagent	Final concentration	Quantity or volume
D-Mannitol (0.6 M)	585 mM	48.75 mL
KCl (2.0 M)	10 mM	0.25 mL
MES (0.5 M, pH 5.7)	10 mM	1.0 mL

Add the reagents to a 50 mL Falcon tube and mix gently by inversion at room temperature. Prepare on the day of the experiment; do not save leftover buffer. Buffer should be stored at room temperature; it expires after 24 h.

**2. MMG buffer**

**Table:** 

Reagent	Final concentration	Quantity or volume
D-Mannitol (0.6 M)	40 mM	33.0 mL
MES (0.5 M)	4 mM	400 μL
MgCl_2_ (1.0 M)	15 mM	750 μL
Sterile ddH_2_O	n/a	15.5 mL

Add the reagents to a 50 mL Falcon tube and mix gently by inversion at room temperature. Avoid making bubbles. Make on the day of the experiment; do not save leftover buffer. Buffer should be stored at room temperature; it expires after 24 h.

**3. W5 buffer**

**Table:** 

Reagent	Final concentration	Quantity or volume
CaCl_2_ (1.0 M)	125 mM	6.25 mL
KCl (2.0 M)	5.0 mM	125 μL
MES (0.5 M, pH 5.7)	0.5 mM	40 μL
NaCl (1.0 M)	1.54 mM	77 μL
Sterile ddH_2_O	n/a	43.5 mL

Add the reagents to a 50 mL Falcon tube and mix gently by inversion at room temperature. Make on the day of the experiment; do not save leftover buffer. Buffer should be stored at room temperature; it expires after 24 h.

**4. WI buffer**

**Table:** 

Reagent	Final concentration	Quantity or volume
D-Mannitol (0.6 M)	500 mM	41.67 mL
MES (0.5 M)	4 mM	400 μL
KCl (2.0 M)	4 mM	100 μL
Sterile ddH_2_O	n/a	7.83 mL

Add the reagents to a 50 mL Falcon tube and mix gently by inversion at room temperature. Make on the day of the experiment; do not save leftover buffer. Buffer should be stored at room temperature; it expires after 24 h.

**5. PEG solution**

**Table:** 

Reagent	Final concentration	Quantity or volume
CaCl_2_ (1.0 M)	100 mM	500 μL
D-Mannitol (1.0 M)	600 mM	3.0 mL
PEG 4000	40% w/v	2.0 g
Sterile ddH_2_O	n/a	Variable
Total		5.0 mL

Add the reagents to a 15 mL Falcon tube and place in a water bath at 55 °C. Allow for PEG to dissolve completely. Make on the day of the experiment; do not save leftover buffer. Buffer should be stored at room temperature; it expires after 24 h.

**6. CTAB buffer**

**Table:** 

Reagent	Final concentration	Quantity or volume
CTAB	2% w/v	2.0 g
EDTA (0.5 M, pH 8.0)	20 mM	4.0 mL
NaCl	1.40 M	8.2 g
Sterile ddH_2_O	n/a	Variable
Tris-HCl (1.0 M, pH 8.0)	100 mM	
Total		100.0 mL

Add the reagents to a 100 mL Falcon tube and mix by inversion at room temperature until completely dissolved. Make on the day of the experiment; do not save leftover buffer. Buffer should be stored at room temperature; it expires after 24 h.

**7. Enzyme suspension**

**Table:** 

Reagent	Final concentration	Quantity or volume
BSA (10%)	0.0985% w/v	100 μL
CaCl_2_ (1 M)	4.93 mM	50 μL
Cellulase	2.9% w/v	300 mg
Macerozyme	0.69% w/v	70 mg
Protoplast buffer (Recipe 1)	n/a	10 mL

Add cellulase, macerozyme, and protoplast buffer to a 50 mL Falcon tube. Incubate at 55 °C for 5 min. Mix by inversion halfway through incubation. Cool on ice for 5 min, then warm to room temperature for 5 min. Add CaCl_2_ and 10% BSA. Mix by inversion. This should be sufficient for 1–2 g of fresh leaf tissue. Make on the day of the experiment; do not save leftover buffer. It should be stored at room temperature; it expires after 24 h.

**Laboratory supplies**

1. Falcon^TM^ 50 mL high clarity conical centrifuge tubes (Fisher Scientific, catalog number 14-432-22)

2. Multi-fold towels (Uline, model number: S-13735)

3. Replacement blades (Havalon, model number: SSC60ADZ)

4. Falcon^®^ 70 μm cell strainer (Fisher Scientific, catalog number: 08-771-2)

5. Eppendorf^TM^ safe-lock 2.0 mL microtube (Fisher Scientific, catalog number: 05-402-11)

6. Eppendorf^TM^ safe-lock 1.5 mL microtube (Fisher Scientific, catalog number: 05-402-27)

7. Corning^TM^ Costar^TM^ 12-well clear TC-treated multiple well plates, individually wrapped, sterile (Fisher Scientific, catalog number: 07-200-82)

8. Corporation bottles 250 mL glass, clear (Fisher Scientific, catalog number: 50-227-0179)

9. Wide-orifice 200 μL pipette tips (Fisher Scientific, catalog number: 50-101-668)

## Equipment

1. Orbital shaker adjustable speed (Onilab, catalog number: SK-O330-M)

2. Refrigerated benchtop centrifuge (Avanti, model: J-15R)

3. Light microscope (Nikon, model: ECLIPSE Si)

4. Neubauer hemocytometer (Superior Marienfeld, catalog number: 0642010)

5. Vortex mixer (Onilab, model: MX-S)

## Software and datasets

1. Cas-Analyzer (Seoul National University College of Medicine, 2016/11)

## Procedure

**A. Growth conditions**

**Figure 1.**
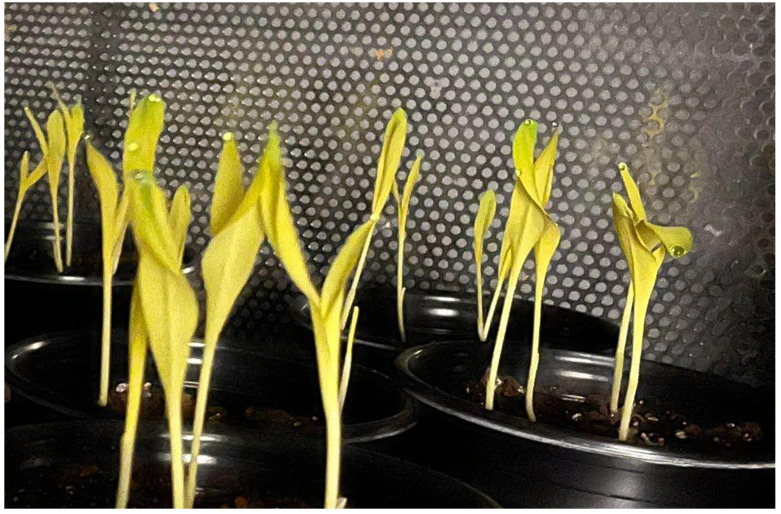
Etiolated maize seedlings grown in dark conditions

1. Germinate seeds between wet paper towels in a closed container for 3 days at room temperature.

2. Transfer germinated seeds to wet soil, with 1–2 seeds in each pot. Grow under a 16-h photoperiod for 3 days.

3. Turn off the lights and grow seedlings under complete darkness for 8 days (Figure 1).

*Note: Seedlings may not need to be watered during this time. Overwatering may negatively impact the health of the plants.*

**B. Protoplast isolation**

*Note: Protoplast isolation relies on enzymatic digestion of the plant cell wall to release intact, wall-free cells. By carefully preparing fresh buffers and enzyme suspensions, protoplasts can be obtained with high yield and viability for downstream applications.*

**Figure 2.**
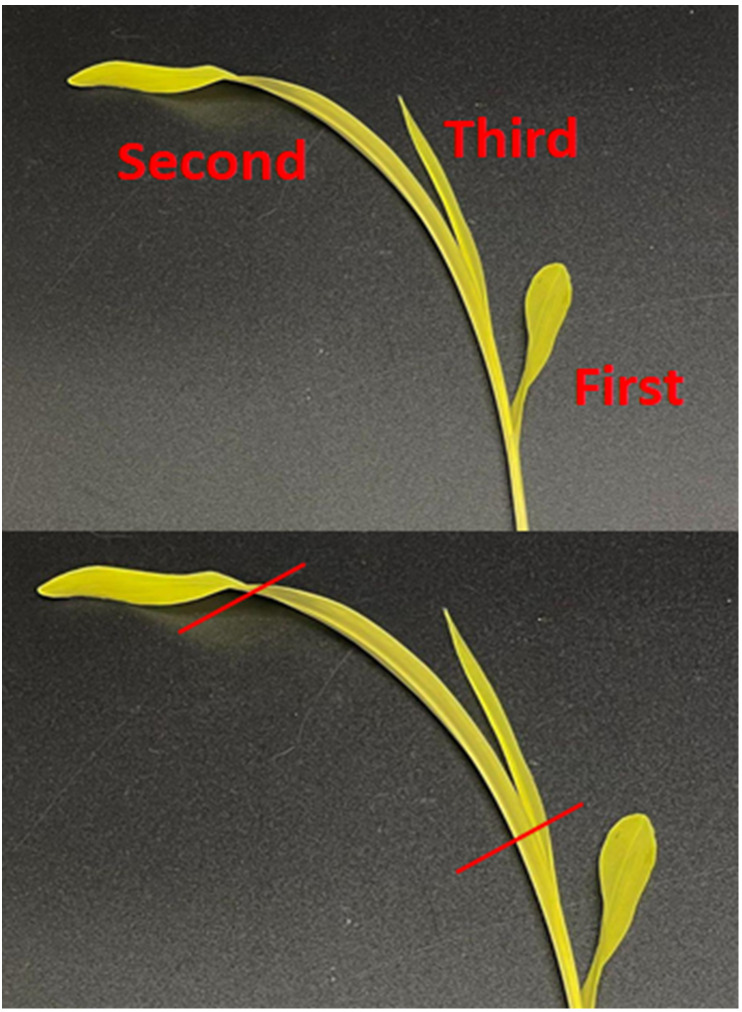
Using only the second leaf, cut off the top and bottom fourth of the leaves

**Figure 3.**
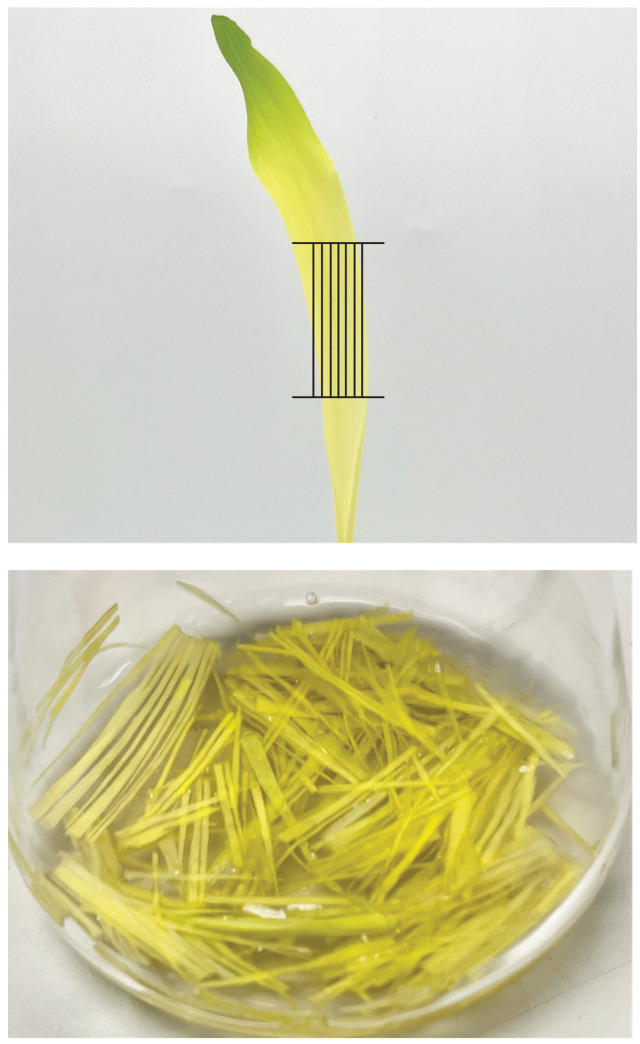
Vertical cutting pattern for maize leaves. Photos are modified from Figure 4 in Higa et al. 2025 [17].

1. Using a new and clean razor blade, cut off the second leaves of each seedling from the base. Cut off and discard the top and bottom fourth of the leaves (Figure 2).

2. Cut the leaves into 2–3 cm pieces before slicing very thin strips (<1–2 mm) in the direction of the leaf veins (Figure 3). Add the leaf strips to a flask with a vacuum arm.

*Note: Make sure not to squeeze/press down hard on the leaves with your fingers at any point while cutting.*

3. Gently add the enzyme suspension to the flask with the leaf strips. Swirl the flask to help the leaf strips submerge in the solution. Vacuum infiltrate the flask for 1 min, twice. You will see bubbles form in the solution. In between vacuums, swirl the flask.

4. Keep in the dark (cover in aluminum foil) and shake at 60 rpm for 3.5 h at room temperature.

5. Shake at 90 rpm for 5 min to release the protoplasts.

6. Gently pour the enzyme suspension containing the protoplasts into a 50 mL Falcon tube using a 70 μm nylon mesh.

*Note: Protoplasts can be fragile, so we recommend carefully pouring the protoplast-containing enzyme solution down the side of the Falcon tube for a gentler approach.*

7. Collect the protoplasts by centrifugation at 200× *g* for 2 min at room temperature. Remove the supernatant by aspiration, being careful not to disturb the protoplast pellet.

*Note: To avoid aspiration of protoplasts, leave a small amount of wash buffer.*

**Figure 4.**
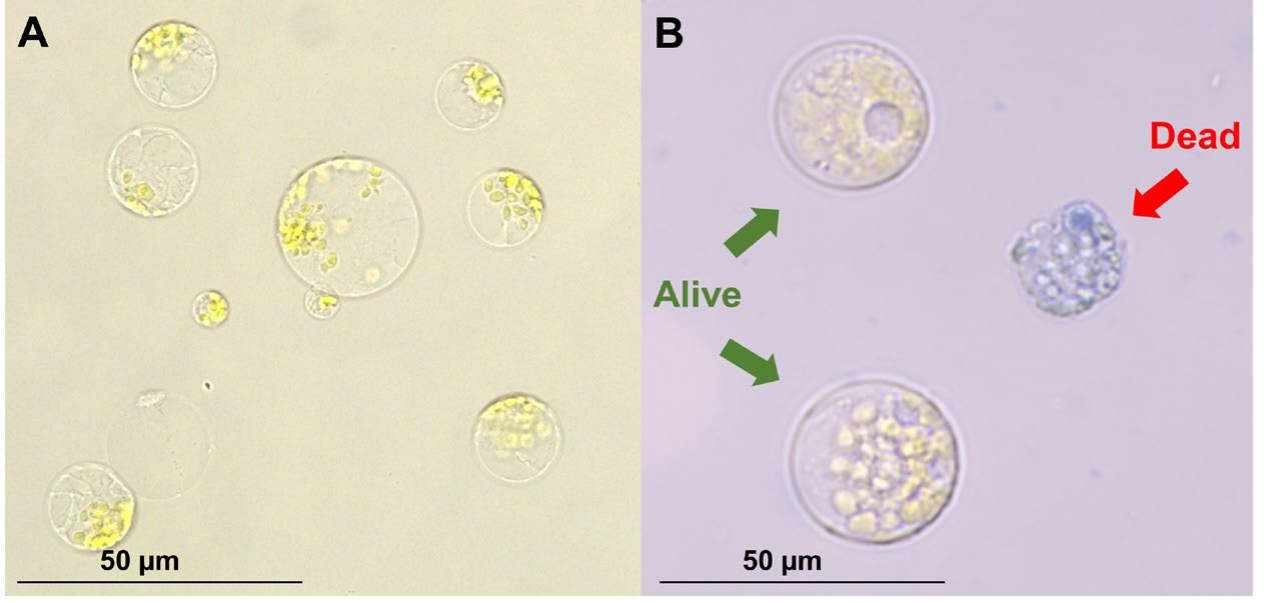
Freshly isolated protoplasts. (A) Protoplasts immediately after filtration and washing. (B) Trypan blue stain, to determine cell viability.

8. Resuspend protoplasts in 10 mL of protoplast buffer and centrifuge at 200× *g* for 2 min. Remove the supernatant by aspiration and repeat the entire wash step.

9. After removing the wash buffer from the protoplast pellet, resuspend in 3 mL of MMG buffer (Figure 4A).

10. Count protoplasts and assess viability using Trypan Blue (0.4%) and a hemocytometer (Figure 4B).

11. Dilute to 500,000 cells/mL using MMG buffer.

**C. Protoplast transfection**

*Note: Protoplast transfection with PEG 4000 enables the direct uptake of plasmid DNA into isolated protoplasts. In the presence of calcium ions, PEG transiently permeabilizes the plasma membrane, facilitating DNA entry. We recommend performing at least three technical replicates to pool for DNA extraction. To ensure high viability, please see General notes 1 and 2.*

1. Mix 20 μL of plasmids (10 μg) with 100 μL of protoplasts in a 2-mL centrifuge tube.

2. Add an equal volume (120 μL) of PEG solution (see Recipe 5). Mix by gently inverting twice.

3. Incubate at room temperature for 15 min.

4. After incubation, add 480 μL of W5 solution to stop the reaction.

5. Centrifuge at 200× *g* for 2 min. Remove supernatant by pipetting.

*Note: The protoplast pellet may be hardly visible. We recommend holding the tube up to a dark colored surface to view the light-colored pellet to ensure you are not disturbing the protoplasts when removing the supernatant.*

6. Wash 1–2 times with 480 μL of WI buffer and centrifuge at 200× *g* for 2 min. Remove supernatant after each wash by pipetting.

*Note: While one wash with WI buffer is sufficient to keep protoplasts alive for 48 h, an additional wash may be beneficial if long-term viability is critical. However, the extra wash may also result in slightly diminished yields.*

7. Resuspend in 200 μL of WI buffer.

8. Add 800 μL of WI buffer to 12-well plates.

9. Transfer the protoplasts resuspended in 200 μL of WI solution into the 12-well plates with 800 μL of WI buffer.

*Note: Using 12-well plates for storage rather than centrifuge tubes markedly increases viability.*

**Figure 5.**
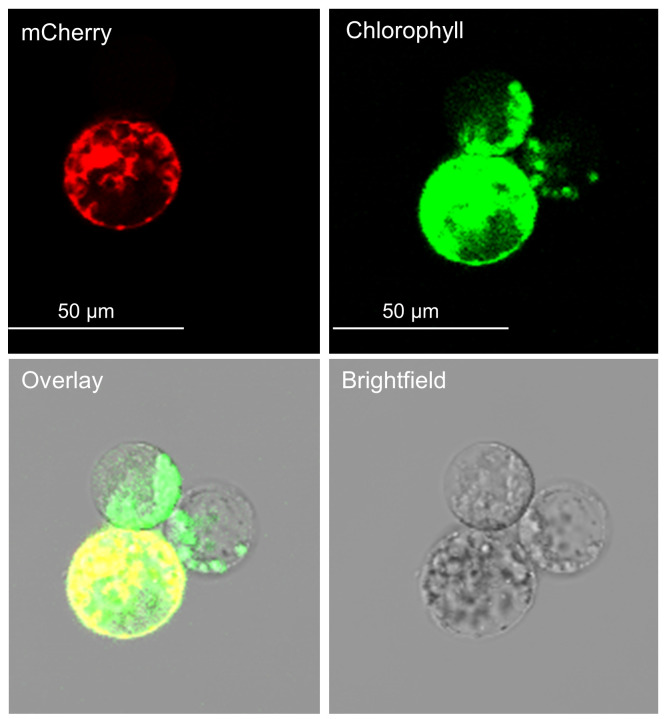
Transfected protoplasts viewed under a confocal microscope. The top-left panel shows mCherry fluorescence, indicating transfected protoplasts. The top-right panel shows chlorophyll autofluorescence, marking all protoplasts. The bottom-right panel shows the brightfield image for cellular morphology and structural context. The bottom-left panel shows a merged image of the brightfield, chlorophyll, and mCherry channels.

10. Incubate at room temperature in the dark overnight.

11. mCherry signal can be observed at 600–650 nm approximately 16 h post-transfection (Figure 5). Confocal or fluorescent microscopy can be used to verify successful transfection and calculate transfection efficiency (transfected protoplasts/total protoplasts). See Troubleshooting (Problem 2) for more information on transfection efficiencies to expect.

**E. DNA extraction**

*Note: DNA extraction from protoplasts can be challenging due to the low concentration of protoplasts in each sample. To maximize yield and ensure sufficient DNA recovery, we use a classic phenol:chloroform extraction instead of commercial kits, which are often less efficient for low-input samples.*

1. After 48 h post-transfection, pool the technical replicates into a 2-mL centrifuge tube. Centrifuge at 3,000× *g* for 2 min to pellet the protoplasts and carefully remove the supernatant. Multiple centrifugation steps may be needed if the volume of pooled replicates exceeds 2 mL.

*Note: The pellet may be difficult to see. We suggest using a dark background to locate the pellet and carefully aspirating the supernatant.*

2. Add 500 μL of CTAB buffer to the sample and vortex. Incubate at 60 °C for 45 min.

3. Vortex the sample again and centrifuge at 17,000× *g* for 10 min. Transfer the supernatant to a new 1.5 mL tube.

4. Add 5 μL of RNase A (10 mg/mL) to the supernatant and incubate for 20 min at 37 °C.

5. Add 1 volume (500 μL) of 25:24:1 phenol:chloroform:isoamyl alcohol to the sample and vortex for at least 30 s. Centrifuge at 17,000× *g* for 10 min.

*Note: Phenol:chloroform:isoamyl alcohol is extremely toxic. Perform all reactions with phenol:chloroform:isoamyl alcohol under a fume hood.*

6. Two phases will form after centrifugation. Transfer the upper aqueous phase into a new tube. The lower phase can be discarded into the appropriate hazardous waste container.

7. Repeat step E5 with the upper aqueous phase.

8. After transferring the next upper aqueous phase to a new tube, repeat step E5 again using just chloroform:isoamyl alcohol to minimize the amount of phenol remaining in the sample.

9. After the final aqueous phase is transferred to a new tube, add 0.7 volumes of ice-cold isopropanol to the sample and briefly vortex.

10. Incubate the samples at -20 °C overnight.

*Note: This can be reduced to just 3 h, but yields may be lower.*

11. After incubation, centrifuge the samples at maximum speed for 15 min at 4 °C. Remove the supernatant, ensuring not to disturb the DNA pellet. Keep the samples on ice.

12. Add 500 μL of ice-cold 70% ethanol to the pellet and centrifuge at maximum speed for 15 min at 4 °C. Carefully remove the supernatant. Perform this wash step twice.

12. Centrifuge the pellet at maximum speed for 5 min at 4 °C. Use a 10 or 20 μL pipette tip to draw up any remaining ethanol, being sure not to disrupt the pellet.

13. Dry the pellet at room temperature for 15 min. When the pellet is completely dry, resuspend the pellet in 20 μL of RNase-free water.

*Note: The pellet may appear as a clear residue, or it may be invisible. The pellet may take slightly longer than 15 min to dry, but be sure not to overdry as this can complicate resuspension of DNA.*

14. Submit DNA for paired-end sequencing. This will be dependent on the method of sequencing used. Briefly, design PCR amplicons so that paired-end reads overlap across the expected edit site. For example, when using 2× 300 bp paired-end sequencing, amplicons of ~300–500 bp should be generated with the anticipated edit positioned near the center of the amplicon, ensuring sufficient read overlap to fully cover the edited region.

## Data analysis

1. Open Cas-Analyzer and select the appropriate analysis mode for paired-end amplicon data.

2. Upload each sample’s Read 1 and Read 2 FASTQ files (experimental and controls).

3. Provide the reference sequence corresponding to the PCR amplicon and enter the gRNA used.

4. Use analysis parameters (recommended starting point; adjust only if justified):

Comparison range (R): 70 (default)

Indicator sequences: auto-defined flanking the cut; allow up to 1 mismatch

Minimum frequency threshold (n): 1 (default)

**Figure 6.**
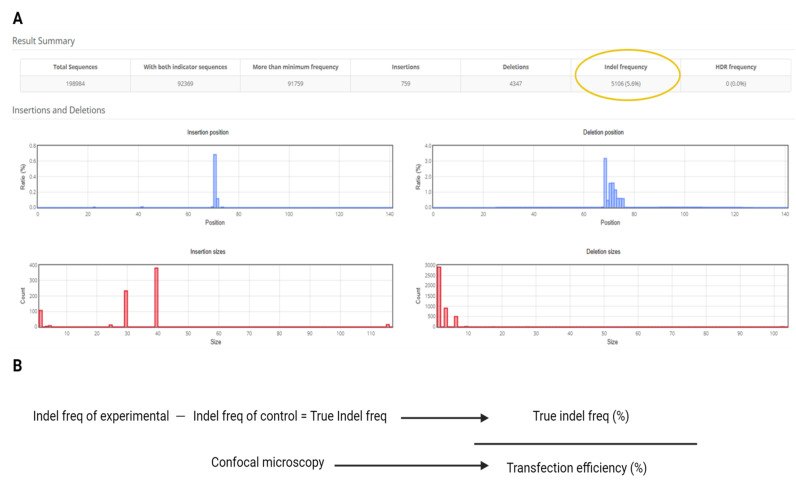
Data analysis. (A) Example of Cas-Analyzer output. (B) Equation for editing efficiency (%) using indel frequency from Cas-Analyzer and transfection efficiency from section C of the Procedure.

5. Run the analysis. Confirm that the read depth and alignment coverage are adequate around the cut site.

6. Record the Indel frequency (%) reported by Cas-Analyzer for each sample (Figure 6A).

7. For each target, obtain Indel% from:

Experimental sample (transfected with Cas9 + gRNA plasmid)

Negative control (water used in place of plasmid)

8. Calculate True Indel%: Indel freq of experimental – Indel freq of negative control (water).

9. Retrieve the Transfection Efficiency% measured for the same replicate set from section C of the Procedure.

10. Compute Editing Efficiency%: Editing efficiency = True indel freq/transfection efficiency.

## Validation of protocol

This protocol (or parts of it) has been used and validated in the following research article(s):

- Higa et al. [2025]. Rapid assessment of CRISPR gRNAs with optimized protoplast transformation in maize. *Plant Cell Reports.*

## General notes and troubleshooting

**General notes**

1. Use wide-orifice tips for all protoplast pipetting steps to prevent damage to protoplasts.

2. As protoplasts can easily lyse, be sure to perform all steps, including pipetting and aspiration, gently.

**Troubleshooting**

**Problem 1**: Low protoplast yield or viability following isolation.

Possible causes: Unhealthy plants, damage to seedling leaves during processing, contaminated/old buffers and solutions, damage to protoplasts during pipetting.

Solutions: Perform all steps gently to prevent protoplast bursting and use wide-orifice tips. Pouring buffers down the side of the tube rather than directly into the protoplast suspension may also help. Prepare all buffers and solutions used for protoplast isolation and transfection on the day of the experiment. Perform isolation and transfection on the same day.

**Problem 2**: Low/no transfected protoplasts.

Possible cause: Larger plasmids may decrease transfection efficiency. This protocol has been tested using plasmids of up to 19 Kb with a transfection efficiency of ~50%.

Solutions: Perform all steps gently to prevent protoplast bursting and use wide-orifice tips. Performing only one wash of WI following transfection can also preserve more protoplasts but may decrease long-term viability.

**Problem 3**: Low/no DNA yield.

Possible causes: Not enough starting material or pellet was removed.

Solutions: Add more technical replicates to the pool for DNA extraction. When removing supernatant, hold the tube to a light source to identify the pellet and avoid disturbing it. Aspirate slowly.
